# Discovery of extracellular vesicle-delivered miR-185-5p in the plasma of patients as an indicator for advanced adenoma and colorectal cancer

**DOI:** 10.1186/s12967-023-04249-6

**Published:** 2023-06-29

**Authors:** Yun-Jie Shi, Yu-Xiang Fang, Tong-Guan Tian, Wei-Ping Chen, Qiang Sun, Fang-Qi Guo, Pi-Qing Gong, Chun-Mei Li, Hao Wang, Zhi-Qian Hu, Xin-Xing Li

**Affiliations:** 1 Department of Anorectal Surgery, Changzheng Hospital, Naval Medical University (Second Military Medical University), Shanghai, 200003 China; 2 Department of General Surgery, Tongji Hospital, Tongji University School of Medicine, Shanghai, 200065 China; 3 Department of Anorectal Surgery, Changhai Hospital, Naval Medical University (Second Military Medical University), Shanghai, 200433 China; 4 State Key Laboratory of Systems Medicine for Cancer, Renji-Med X Clinical Stem Cell Research Center, Renji Hospital, School of Medicine, Shanghai Jiao Tong University, Shanghai, 200127 China; 5 Shanghai Key Laboratory of Signaling and Disease Research, Frontier Science Center for Stem Cell Research, School of Life Sciences and Technology, Tongji University, Shanghai, 200120 China; 6 Institute of Basic Medicine and Cancer (IBMC), Zhejiang Cancer Hospital, Chinese Academy of Sciences, Hangzhou, 310022 Zhejiang China; 7 Department of Gastrointestinal Surgery, Changzheng Hospital, Naval Medical University (Second Military Medical University), Shanghai, 200003 China; 8 Department of Ultrasound, Shanghai Fourth People’ Hospital, School of Medicine, Tongji University, Shanghai, China

**Keywords:** Extracellular vesicle, microRNA, Colorectal advanced adenoma, Colorectal cancer, Liquid biopsy

## Abstract

**Background:**

We aimed to evaluate whether extracellular vesicles (EV)-derived microRNAs (miRNAs) can be used as biomarkers for advanced adenoma (AA) and colorectal cancer (CRC).

**Methods:**

We detected the changes in the plasma EV-delivered miRNA profiles in healthy donor (HD), AA patient, and I-II stage CRC patient groups using miRNA deep sequencing assay. We performed the TaqMan miRNA assay using 173 plasma samples (two independent cohorts) from HDs, AA patients, and CRC patients to identify the candidate miRNA(s). The accuracy of candidate miRNA(s) in diagnosing AA and CRC was determined using the area under the receiver-operating characteristic curve (AUC) values. Logistic regression analysis was performed to evaluate the association of candidate miRNA(s) as an independent factor for the diagnosis of AA and CRC. The role of candidate miRNA(s) in the malignant progression of CRC was explored using functional assays.

**Results:**

We screened and identified four prospective EV-delivered miRNAs, including miR-185-5p, which were significantly upregulated or downregulated in AA vs. HD and CRC vs. AA groups. In two independent cohorts, miR-185-5p was the best potential biomarker with the AUCs of 0.737 (Cohort I) and 0.720 (Cohort II) for AA vs. HD diagnosis, 0.887 (Cohort I) and 0.803 (Cohort II) for CRC vs. HD diagnosis, and 0.700 (Cohort I) and 0.631 (Cohort II) for CRC vs. AA diagnosis. Finally, we demonstrated that the upregulated expression of miR-185-5p promoted the malignant progression of CRC.

**Conclusion:**

EV-delivered miR-185-5p in the plasma of patients is a promising diagnostic biomarker for colorectal AA and CRC.

*Trial registration* The study protocol was approved by the Ethics Committee of Changzheng Hospital, Naval Medical University, China (Ethics No. 2022SL005, Registration No. of China Clinical Trial Registration Center: ChiCTR220061592).

**Supplementary Information:**

The online version contains supplementary material available at 10.1186/s12967-023-04249-6.

## Introduction

Colorectal cancer (CRC) can develop from adenoma, serrated polyps, or chronic inflammation caused by inflammatory bowel disease. The transformation from adenoma to carcinoma is the main cause of CRC tumorigenesis [1]. In this process, the gradual accumulation of oncogenes and epigenetic changes within cells promotes the transformation of the normal intestinal epithelium to early-stage adenoma, advanced adenoma (AA), and even cancer. AA refers to an adenoma that is 10 mm or greater in diameter or has high-grade dysplasia, or shows villous histology (> 25% villous component), or contains intramucosal carcinoma [2]. AA is more likely to develop into CRC, and early removal of an AA can reduce the incidence and mortality of CRC [3]. The late diagnosis of CRC is one of the main factors contributing to the lower survival rates and mortality of patients with CRC worldwide [4]. The 5-year survival rate of patients with early-mid stage CRC is > 90%. In contrast, the median survival of patients with advanced CRC patients and distant metastasis is < 2 years [5]. Therefore, screening for AA and early-mid stage CRC is the key to reducing the incidence and mortality of CRC. However, specific biomarkers, especially those continuously tracing markers, were still limited which could be used to detect a continuous profiling change during the process from healthy donors (HD) to AA and in turn to CRC.

To date, carcinoembryonic antigen (CEA) and carbohydrate antigen 199 (CA199) are the specific biomarkers for the diagnosis of AA in clinical settings and are the most common hematological biomarkers for the auxiliary diagnosis of CRC. Although serum CEA and CA199 levels show some diagnostic value in patients with advanced CRC (stages III–IV) [6], their diagnostic value is low for AA and early-mid stage CRC (stages I–II) [7]. Therefore, CEA and CA199 cannot be used as continuously tracing markers for the diagnosis of AA and CRC.

Extracellular vesicles (EVs) have become important liquid biopsy tools in recent years [8]. EVs can be extracted from several body fluids, including blood, urine, saliva, and cerebrospinal fluid [9]. It is a small vesicle with a diameter of 30–1000 nm, and the two major types include exosome (30–100 nm diameter) and microvesicle (100–1000 nm diameter). EVs, generated by all cells, can deliver multiple miRNA, lncRNA, DNA fragments, and proteins as stable cargos enveloped in their lipid bilayer membranes [10, 11]. Therefore, the expression of EV-delivered miRNAs can be used as potential biomarkers for the diagnosis of cancer and monitoring the progress of the disease [12]. The expression of EV-delivered noncoding RNA is higher in patients with gastrointestinal cancer than that in normal volunteers, and EV-derived miRNAs are more sensitive markers than CEA and CA199 for diagnosing early-stage tumors [13, 14]. However, EV-delivered miRNAs that can diagnose both AA and CRC still need to be identified.

Here, we attempted to screen and identify new plasma EV-delivered miRNAs, which can be used as biomarkers to diagnose both AA and CRC. Our results indicated that miR-185-5p might be a useful potential biomarker.

## Materials and methods

### Clinical sample collection

The study protocol was approved by the Ethics Committee of Changzheng Hospital, Naval Medical University, China (Ethics No. 2022SL005, Registration No. of China Clinical Trial Registration Center: ChiCTR220061592). All procedures involving human participants were performed at the Changzheng Hospital following the ethical standards of the institution as well as the 1964 Helsinki Declaration and its subsequent amendments [15]. We obtained written informed consent from participants.

The inclusion criteria were: (1) age 20–80 years; (2) Karnofsky score ≥ 80 [16]; (3) gastroscopy performed within 3 months, and no obvious lesions detected (including malignant lesions, polyps, and ulcers); (4) written informed consent of patients and their families. The exclusion criteria were: (1) patients with American Society of Anesthesiologists physical status IV to V [17]; (2) pregnant or lactating women; (3) serious cardiovascular disease, uncontrollable infection, or other uncontrollable concomitant diseases; (4) other multiple tumor disease history or other genetic diseases.

Healthy donor (HD) samples were the whole blood samples collected from patients who underwent electronic colonoscopy and colorectal lesions were not found. Colorectal polyp samples were the whole blood samples collected from patients who underwent electronic colonoscopy and ≥ 10 mm colorectal polyps were found. CRC samples were the whole blood samples collected from patients with CRC who were not undergoing new adjuvant treatment in Changzheng Hospital and pathological symptoms were confirmed using endoscopic biopsy and imaging examination before surgery. Venous blood samples (6 mL) were collected in an EDTA tube (Becton Dickinson, New Jersey, USA). The blood samples were centrifugated (3000 g, 15 min,4 °C) to separate the plasma. The plasma samples were grouped into HD (n = 50), AA (n = 71), and CRC (n = 70) groups based on the pathological findings and stored at − 80 °C. Age of HD, AA, and CRC groups were matched.

CRC tumor and paracancerous tissue samples were collected from surgical resection specimens of CRC patients. These patients were not undergoing new adjuvant treatment in Changzheng Hospital and pathological symptoms were confirmed using endoscopic biopsy and imaging examination before surgery.

### EV extraction and purification from plasma and cells

One milliliter of plasma was used to isolate EVs corresponding to each sample. The plasma samples were thawed in a 37 °C water bath and centrifuged at 20–30 °C at 10000 g for 30 min to eliminate residual cell fragments. The plasma EVs were extracted and purified using the plasma exosome extraction and purification kit (Cat. No. EZB-exo1, EZBioscience, California, USA) according to the manufacturer’s protocol. Exosome Isolation Kit (from cell culture media; Cat. No. EZB-exo2, EZBioscience) was used to extract and purify the cell culture media EVs according to the manufacturer’s protocol. Briefly, the cell lines were cultured with depleted fetal bovine serum (FBS) media for 24 h. 20 mL cell culture media were harvested and centrifugated at 3000 g for 10 min at 4 °C to remove cells and debris. Cell-free culture media were transferred to a new tube and added 5 mL Exosome Precipitation Reagent (EPR). The culture media/reagent mixture were incubated at 2–8 °C overnight. After incubation, the sample was centrifuged at 10000 g for 30 min at 4 °C. Discard the supernatant, appropriate 1 × PBS resuspend the EVs. The exosomes from culture media were identified by transmission electron microscopy.

### Transmission electron microscopy (TEM)

The plasma/cell culture media EV protein concentration was determined using the BCA Protein Assay kit (Beyotime, Shanghai, China). The protein concentration was set at < 0.5 mg/mL (200-fold dilution of the initial sample) to obtain a relatively clean background field under the TEM. Approximately 20 μL EVs were dropped on the clean surface of the sealing membrane. The copper mesh was placed on the EV droplet, and the complete assembly was transferred to 1% uranyl acetate negative dye solution for 10 min in dark and slowly dried using filter paper. Finally, after drying under the incandescent lamp, EVs were observed using a TEM (JEOL-JEM1400, Tokyo, Japan) for electron microscope photos at 80 kV. Digital Micrograph software (Gatan, California, USA) was used for TEM data acquisition and analysis.

### Nanoparticle tracking analysis (NTA)

Plasma EV samples were detected using the Zetaview PMX110 nanoparticle analyzer (Particle Metrix, Meerbusch, Germany) and analyzed using the ZetaView 8.04.02 software. Briefly, EV suspension with concentrations between 1 × 10^7^/mL and 1 × 10^9^/mL was examined using the ZetaView PMX 110 equipped with a 405 nm laser to determine the size and quantity of particles isolated. A video of 60-s duration was taken with a frame rate of 30 frames/sec, and particle movement was analyzed using the ZetaView 8.04.02 software.

### Western blotting (WB)

EV samples were lysed using RIPA buffer (Beyotime) with 1 × Protease Inhibitor Cocktail (Beyotime) and incubated on ice for 30 min to isolate total protein. After incubation, the sample was centrifuged at 12000 rpm for 30 min at 4 °C. Obtain the supernatant, the EV protein concentration was determined using the BCA Protein Assay kit (Beyotime). The absorbance at 562 nm was analyzed by a microplate reader (BMG LABTECH, Offenburg, Germany), and the protein concentration of the samples was calculated by plotting the standard curve. 1 × SDS Loading Buffer (Beyotime) was used to dilute all samples to a uniform protein concentration. The samples were boiled in a metal bath at 100 °C for 20 min, followed by centrifugation and shaking. The samples (equivalent to 20 μg protein) were separated on 4–12% SDS-PAGE and then transferred to a 0.2-μm-pore PVDF membrane (Millennium, New Jersey, USA). The membranes were blocked with 5% BSA for 1 h at room temperature and incubated with primary antibodies at 4 °C overnight. We used primary antibodies against EV-specific markers CD9 (dilute concentration 1:1000, Abcam, Cat. No. ab223052, Cambridge, England), CD63 (dilute concentration 1:1000, Abcam, Cat. No. ab271286), and ALIX (dilute concentration 1:1000, Abcam, Cat. No. ab186429). The membranes were washed by TBST and incubated with an HRP-linked secondary antibody (dilute concentration 1:8000, Abcam, Cat. No. ab288151) for 1 h at room temperature. The membranes were developed using an HRP substrate and photographed using an ECL detection apparatus (Thermo Fisher Scientific, Massachusetts, USA).

### miRNA deep sequencing assay

The plasma EVs were extracted and purified using the plasma exosome extraction and purification kit (Cat. No. EZB-exo1, EZBioscience, California, USA) according to the manufacturer's protocol. The total RNA or purified miRNA fragments of EV samples were extracted and reverse transcribed into cDNA using Reverse Transcription Kit (Ambion, New Jersey, USA), and then amplified using PCR kit (Takara, Tokyo, Japan) following the manufacturer’s instructions. Further, the miRNA libraries were constructed using TruSeq Stranded RNA LTSample Prep Kit (Illumina, San Diego, CA, USA) according to the manufacturer’s instructions, and the qualified library was sequenced. Agilent 2100 Bioanalyzer (Agilent, California, USA) was used to check the size and purity of the library. The raw data (raw reads) was obtained from an Illumina HiSeqTM 2500 sequencing platform. The information analysis process was as follows. The connectors at both ends of the reads, the reads with fragment lengths less than 17nt, and low-quality reads were removed to complete the preliminary data filtering and obtain high-quality data (clean reads). C +  + and R languages were used to control the quality of raw data and obtain clean and high-quality data. The miRNA expression was analyzed using the Perl software, and the differential expression was obtained using the edgeR software. All the tests, heat map, KEGG, and GO drawing were performed by Ribobio Comp (www.ribobio.com; Guangzhou, Guangdong, China). The raw data of miRNA deep sequencing assay obtained in this study are available in the Gene Expression Omnibus (GEO) database with accession number GSE220445. The threshold of significantly different miRNAs was set at fold change > 1.5 or < 0.67 and P < 0.05.

### RNA extraction and quantitative real-time PCR for plasma EVs

Real-time PCR was used for validation of EV-delivered miRNA relative expression. Exo-RNA purification Kit (Cat. No. EZB-exo-RN1, EZBioscience) was used to extract and purify plasma EV-derived total RNA from plasma samples according to the manufacturer’s protocol. In the process of extracting and purifying plasma EV-derived total RNA, after plasma EVs were fully lysed by the lysis buffer, a *Caenorhabditis elegans*-specific microRNA mimic cel-miR-39-3p (100 pM; Thermo Fisher Scientific) was added as an exogenous control into the samples for the TaqMan MicroRNA Assay [18]. An Agilent 2100 Bioanalyzer (Agilent, California, USA) was used to quantify the same RNA amount of EV samples for the RT-qPCR reaction. All candidate EV-derived miRNAs and exogenous control mimic cel-miR-39-3p were reversed transcribed using TaqMan™ advanced miRNA cDNA Synthesis Kit (Cat. No. A28007, Thermo Fisher Scientific). TaqMan MicroRNA Assay (Thermo Fisher Scientific) was used to measure the relative expression of candidate miRNAs. The catalog numbers of specific miRNA probes were: has-miR-185-5p: A25576/477939, has-miR-30d-5p: A25576/478606, has-miR-126-3p probe: A25576/477887, has-miR-4433b-3p probe: A25576/479802, and mimic cel-miR-39-3p: A25576/478293 (Thermo Fisher Scientific). We used a 7500 Fast PCR instrument (Thermo Fisher Scientific) to perform the RT-qPCR assay.

### RNA extraction and quantitative real-time PCR for tissue and cells

The tissue RNA was extracted using Trizol (Thermo Fisher Scientific) method. Approximately 800 µL Trizol was added into the sample in a 1.5 mL centrifuge tube to grind tissue, and then 1/5 volume of chloroform was added. The samples were centrifuged at 4 °C and 12000 rpm for 15 min and isopropanol was added to precipitate RNA. Then, precipitated samples were washed using 75% ethanol, centrifuged at 4 °C and 12000 rpm for 5 min, and supernatant was discarded to obtain the pellet. The lid was kept open to dry the white precipitate until it was transparent, and an appropriate amount of distilled water was added to dissolve RNA. EZ-press RNA Purification Kit (Cat. No. B0004D, EZBioscience) was used to extract cell RNA according to the standard protocol. Nanodrop2000 (Thermo Fisher Scientific) was used to quantify the same RNA amount for the qPCR-RT-reaction. The MiDETECT A TrackTM miRNA qRT-PCR Starter Kit (Cat. No. R11068.5, Ribobio) was used to perform poly A tailing, reverse transcriptional reaction, and qPCR following the manufacturer’s protocol. A 7500 Fast PCR instrument (Thermo Fisher Scientific) was used to perform the RT-qPCR. The U6 RNA level was used as an internal control for data normalization. The forward primer of miR-185-5p was 5'-CCACTCTATGGAGAGAAAGGCAG-3'.

### Cell culture

Human colonic epithelial cell line FHC and CRC derived cell lines (SW480, CaCo-2, HCT116 and RKO) were purchased from the Type Culture Collection of the Chinese Academy of Sciences (Shanghai, China) and maintained in the Dulbecco’s Modified Eagle Medium (DMEM) medium containing 10% FBS (Gibco, USA) at 37 °C in 5% CO_2_.

### Cell transfection

Cells were seeded into 96-well plates (3 × 10^3^ cells/well) or 24-well plates (2 × 10^5^ cells/well) for cell transfection. Transfection can be performed when the cell confluence exceeded 80–90%. miR-185-5p overexpression was achieved by transfecting cells with 100 nM miR-185-5p mimics (RiboBio), whereas knockdown was achieved by transfecting the cells with 100 nM miR-185-5p inhibitors (RiboBio), and Lipofectamine 2000 (Invitrogen, USA) was used for cell transfection according to the manufacturer’s protocol. 6 h later, the cells were replaced with DMEM medium containing 10% FBS and cultured for 24 h for subsequent CCK-8 assay (96-well plates) or transwell assay (24-well plates). The abovementioned gene sequences were:miR-185-5p mimics: 5'-UGGAGAAAGGCAGUUCCUGA-3'miR-185-5p inhibitors: 5'-ACCUCUUUCCGUCAAGGACU-3'.

### Lentiviral vector infection

Lentiviral vectors containing miR-185-5p mimics and relevant controls were constructed and packaged into lentivirus by Genomeditech Comp (Shanghai, China). Approximately 1.5 × 10^5^ SW480 cells were infected with related lentivirus (MOI = 10) along with 10 μg/mL polybrene dissolved in the culture medium. After 24 h of incubation, the culture supernatant was replaced with fresh medium containing 10% FBS and puromycin (0.5 μg/ml, Maryland, USA) to select for cells that were successfully transduced. The stable overexpression of miR-185-5p (5'-UGGAGAAAGGCAGUUCCUGA-3') was confirmed using qPCR. The stable cell was used in animal experiment.

### Cell proliferation assay

Cells were seeded into 6-well plates (1 × 10^5^ cells/well) and cultured for 48 h. The medium was discarded, cells were fixed with 4% paraformaldehyde and dyed with 0.1% crystal violet, and images were obtained using a digital camera (Olympus, Tokyo, Japan) to record the results.

### CCK-8 assay

Cells were seeded into 96-well plates (3 × 10^3^ cells/well) and cultured overnight. After 24 h transfection, the medium was removed and CCK-8 reagent (Targetmol, Massachusetts, USA) was added at 10% concentration. The absorbance was measured using a microplate reader (BioTek, Vermont, USA) at 450 nm.

### Cell migration assay

The cell transwell assay was performed to assess cell migration. The transwell assay was performed using 6.5-mm transwell chambers having 8-μm pores (Costar, New York State, USA). Cells were seeded into the upper insert at 2 × 10^5^ cells/well and cultured overnight. After 24 h transfection, the upper and lower chambers were replaced with serum-free medium and 20% fresh medium, respectively. After 36 h of incubation, the upper surface of each membrane was cleaned, and cells adhered to the insert surface were fixed and stained with 0.1% crystal violet.

### Animal experiments

The miR-185-5p-overexpressing SW480 cells were subcutaneously implanted into the right side of the armpit under the skin in 5-week-old BALB/c-nu nude mice (5 × 10^6^ cells/mouse). After tumorigenesis, the tumor size was recorded every four days, and the tumor volume was calculated using the formula V = 1/2 × a × b^2^ (where a and b are the longest and shortest diameters, respectively).

### Histological studies

The tumor tissues were dehydrated using an ascending series of ethanol solutions, cleared using xylene, and embedded in paraffin wax. The embedded tissues were cut into thin Sects. (2–7 µm) using a rotatory microtome. The Ki67 Kit (Merck, Cat. No. SAB5700770, New Jersey, USA) was used to assess the proliferative capacity of intestinal epithelial crypt cells. The Tunel Kit (Merck, Cat. No. APT110) was used to determine the apoptosis of intestinal epithelial crypt cells. The images were captured under a fluorescence microscope (Olympus).

### Statistical analysis

We used the 2^−ΔΔ Ct^ method to calculate the relative expression of the candidate miRNAs. Area under the receiver-operating characteristic curve (AUC) values were used to quantify the diagnostic efficacy of candidate miRNAs for AA and CRC. We used the Delong test to compare the significance of AUC between candidate miRNAs and CEA/CA199, and the MedCalc software was used to analyze the data. A logistic regression analysis was performed using the SPSS 21.0 software to evaluate the diagnostic association of candidate miRNAs with AA and CRC by chi-square test. The measurement data were compared by independent t-test or analysis of variance using the GraphPad 8.0 software. P-values of < 0.05 were considered statistically significant.

## Results

### EV-delivered miRNAs are dysregulated in AA and CRC

We extracted and purified EVs from 18 plasma samples (obtained from 6 HDs, 6 patients with AA, and 6 patients with I-II stage CRC) to determine the biological characteristics of EVs. There were no differences in purified EVs from plasma samples of HD, AA, and CRC group. The clinical and pathologic characteristics of the patients are summarized in Additional file 7: Table S1. The morphological features, surface markers, and particle size distribution of EVs were evaluated using TEM, WB, and NTA respectively. TEM images exhibited a similar pattern among the three groups (Additional file 1: Fig.S1A). The expression of major EV surface positive markers such as CD9, CD63, and ALIX was also similar among the three groups (Additional file 1: Fig.S1B). Moreover, we also checked the expression of negative EV marker Calnexin among the three groups. As expected, the expression of Calnexin was not observed in plasma derived EVs (Additional file 1: Fig.S1B). Consistently, NTA revealed similar characteristics among HD, AA, and CRC groups for average EV size and concentration (Additional file 1: Fig. S1C, D). Moreover, the total RNA concentration in plasma or RNA concentration in plasma-derived EVs was not significantly different among the three groups (Additional file 1: Fig.S1E). Taken together, these results suggested that the biological characteristics of plasma EVs and EV-delivered total RNA concentration were similar in HD, AA, and CRC groups.

**Fig. 1:**
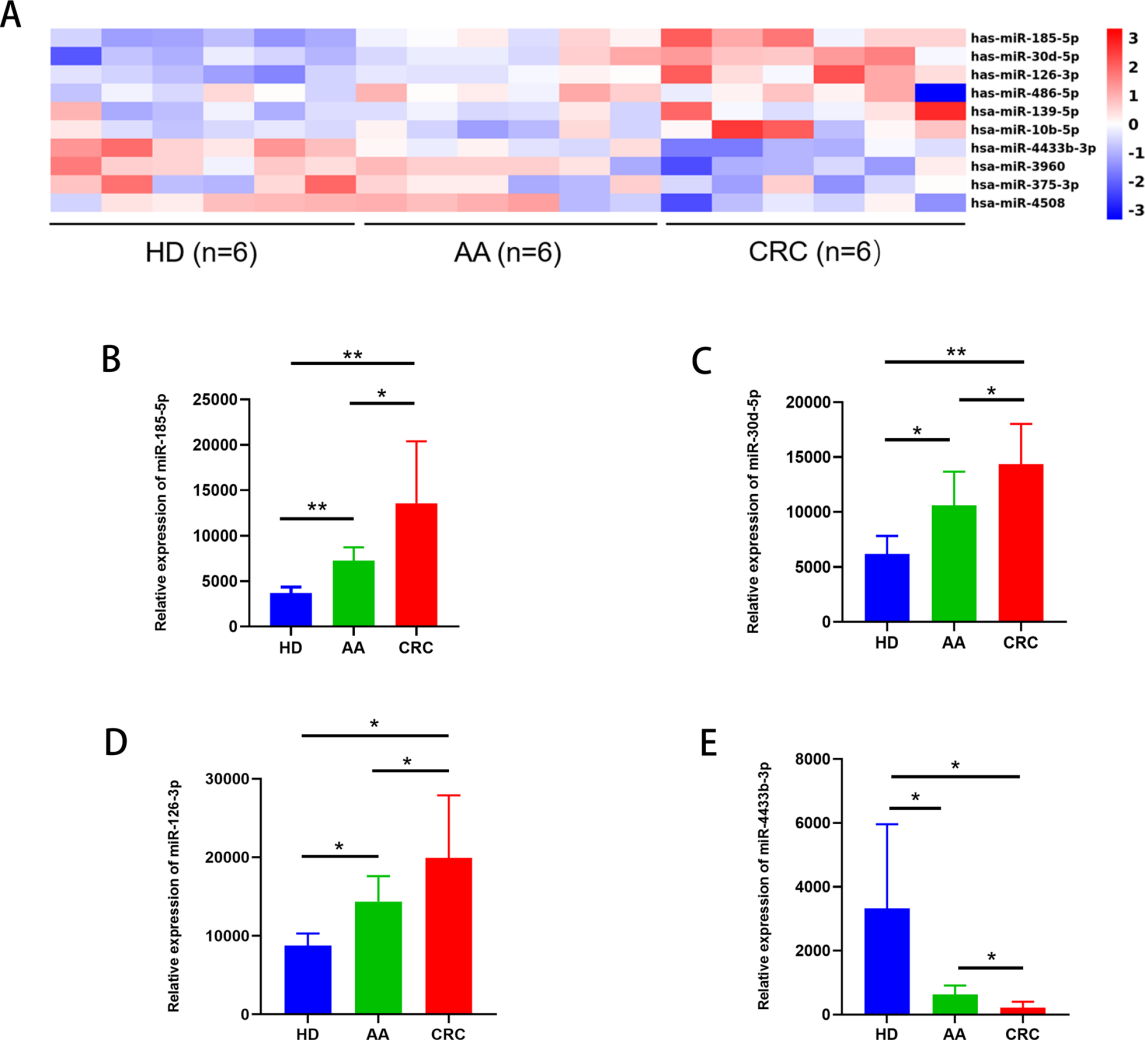
Screening of four candidate EV-delivered miRNAs from plasma. **A** Heatmap showing the top 10 differentially expressed miRNAs showing an increasing or decreasing trend from HD to AA and further to CRC. **B**–**E** Mean relative expression of plasma EV-delivered miR-185-5p, miR-30d-5p, miR-126-3p, and miR-4433b-3p in HD, AA, and CRC groups. *, P < 0.05; **, P < 0.01

Next, total RNA was used for miRNA profiling using miRNA deep sequencing. We analyzed differential expressions of miRNAs between HD and AA, AA and CRC, and HD and CRC groups. Compared with the HD group, we found that 24 and 33 miRNAs were significantly upregulated and downregulated in the AA group, respectively, and 60 and 27 miRNAs were significantly upregulated and downregulated in the CRC group, respectively. Compared with the AA group, 41 and 85 miRNAs were significantly upregulated and downregulated in the CRC group, respectively. By ordering the absolute value of log2 (Fold change) and P value, we selected the top 25 different miRNAs in each comparison for cluster analysis (Additional file 2: Fig.S2). To screen for biological markers that could be used for the continuous diagnosis of colorectal AA and CRC, reflecting the whole process of disease progression from the normal state to AA and further to CRC, our preliminary screening identified 10 candidate miRNAs that were upregulated or downregulated in both AA vs. HD and CRC vs. AA groups (Fig. 1A and Additional file 8: Table S2). Among these miRNAs, we selected three significantly upregulated miRNAs (top 3/6), namely miR-185-5p, miR-126-3p, and miR-30d-5p (fold change > 1.5, P < 0.05 in both AA vs. HD and CRC vs. AA groups) (Fig. 1B–D) and a downregulated miRNA miR-4433b-3p (top 1/4; fold change < 0.67, P < 0.05 in both AA vs. HD and CRC vs. AA groups) (Additional file 1: Fig. 1E) for further analysis to determine whether these four miRNAs can be used as biomarkers for the diagnosis of both AA and CRC.

### Validation of EV-delivered miRNAs as biomarkers for AA and CRC

**Fig. 2:**
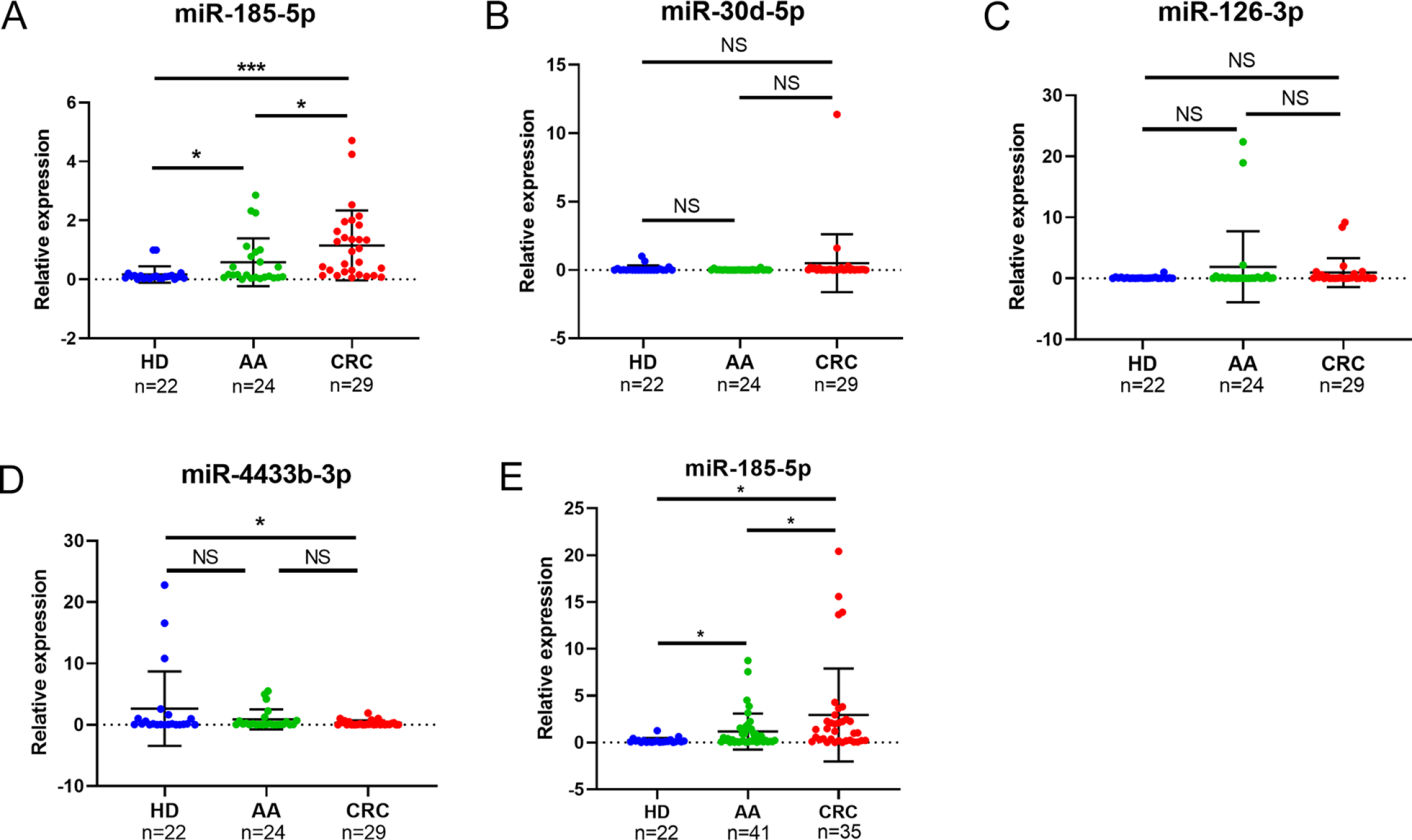
Validation of EV-delivered miRNAs as biomarkers for AA and CRC. Relative expression of plasma EV-delivered **A** miR-185-5p **B** miR-30d-5p **C** miR-126-3p **D** miR-4433b-3p among HD, AA, and CRC patients in Cohort I. **E** Relative expression of plasma EV-delivered miR-185-5p among HD, AA, and CRC patients in Cohort II. Horizontal lines represent the mean ± SD of data in each group. The expression of each miRNA was normalized relative to the expression of reference miRNA cel-miR-39-3p (*C. elegans*-specific microRNA mimic as an exogenous control). *, P < 0.05; ***, P < 0.001; *NS* non-significance

We attempted to evaluate the diagnostic value of the candidate miRNAs for AA and early-mid stage CRC. Therefore, we validated their expression in two independent cohorts (Additional file 3: Fig.S3). In Cohort I (including 22 HD, 24 AA, and 29 stages I-II CRC samples), the expression of miR-185-5p in the AA and CRC groups was significantly higher than in the HD group. Furthermore, its expression in the I-II stage CRC group was also significantly higher than that in the AA group (Fig. 2A), which indicated a continuous upregulation of this miRNA during tumorigenesis. In contrast, miR-30d-5p and miR-126-3p were not significantly upregulated in the CRC/AA group compared with that in the HD group (Fig. 2B, C). Although miR-4433b-3p expression was significantly downregulated in the CRC group compared with that in the HD group, it was similar between HD and AA and AA and CRC groups (Fig. 2D). Overall, these results indicated that EV-delivered miR-185-5p was a potential biomarker for the diagnosis of AA and I-II stage CRC.

The circulating biomarkers that can be used to diagnose AA, I-II stage CRC (i.e., CRC tumorigenesis), and advanced CRC (i.e., CRC progression) are limited. Therefore, to investigate whether miR-185-5p could also be an available biomarker for advanced CRC, we constructed Cohort II, including 22 HD, 41 AA, and 35 stages III-IV CRC samples, for further validation. Consistent with the results observed in Cohort I, we found that the expression of miR-185-5p in the AA group was significantly higher than that in the HD group. Interestingly, compared with that in the HD/AA group, the expression of miR-185-5p was significantly increased in the advanced CRC group, which indicated that the expression of miR-185-5p was stably upregulated during disease progression from tumorigenesis (stages I–II) to advanced stages (stages III-IV) (Fig. 2E, Additional file 4: Fig. S4). Taken together, our results indicated that miR-185-5p could serve as a biomarker for continuously tracing the development of the disease from precancerous lesions (AA) to tumorigenesis (CRC stages I–II) and further to tumor progression (CRC stages III–IV).

### EV-delivered miR-185-5p is a potential biomarker for colorectal AA

**Fig. 3:**
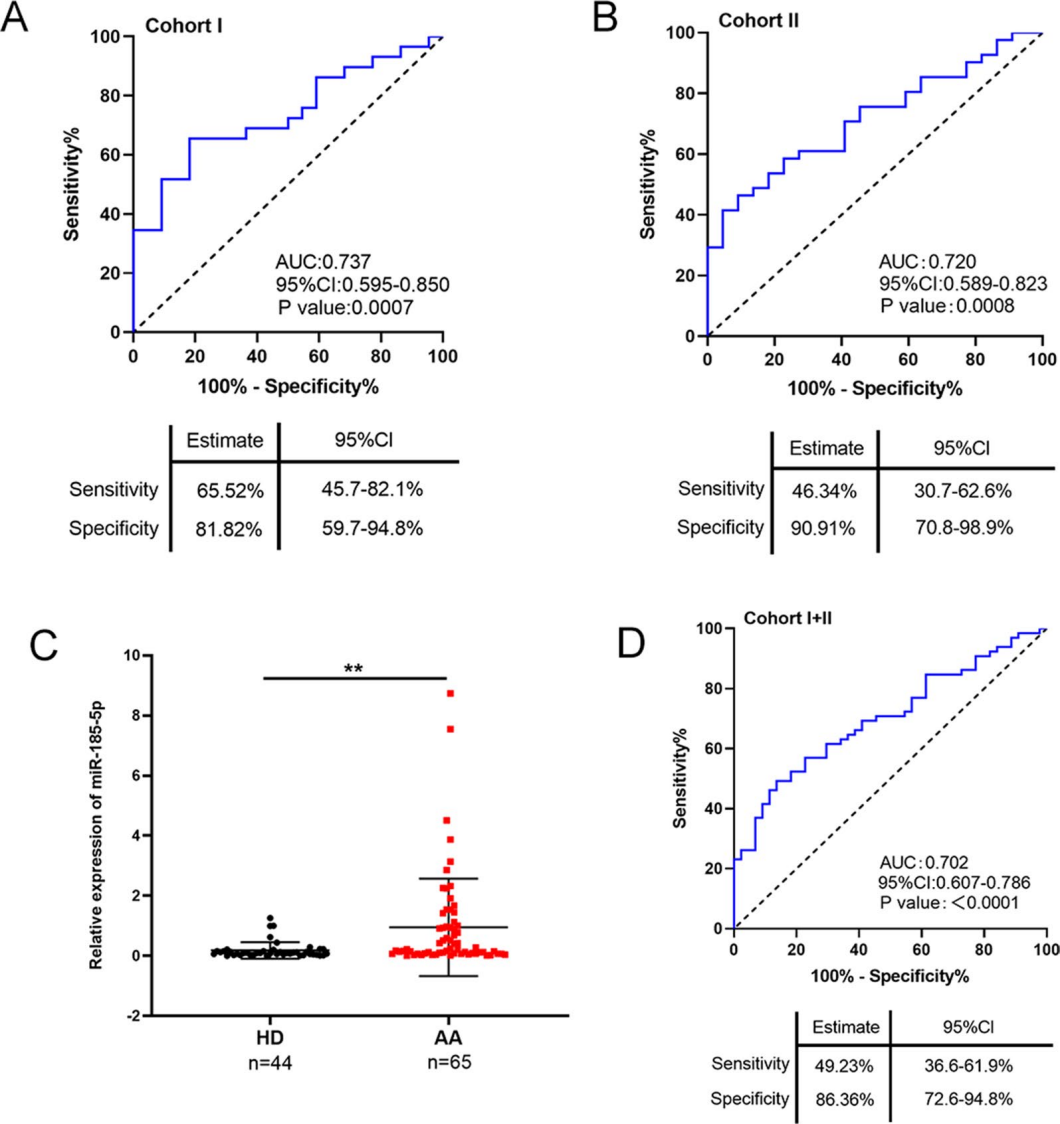
EV-delivered miR-185-5p is a potential biomarker for colorectal AA. **A** ROC curve analysis of EV-delivered miR-185-5p as a parameter to discriminate AA from HD in Cohort I. **B** ROC curve analysis of EV-delivered miR-185-5p as a parameter to discriminate AA from HD in Cohort II. **C** Relative expression of plasma EV-delivered miR-185-5p in HD and AA patients in the whole cohort (I + II). **D** ROC curve analysis of EV-delivered miR-185-5p as a parameter to discriminate AA from HD in the whole cohort (I + II). **, P < 0.01

We further evaluated the efficacy of miR-185-5p in the diagnosis of AA by analyzing AUC values. In Cohort I, the AUC value of miR-185-5p for the diagnosis of AA was 0.737 with 65.52% sensitivity and 81.82% specificity (Fig. 3A). Consistently, the results in Cohort II showed that the AUC value of miR-185-5p for the diagnosis of AA was 0.720 with 46.34% sensitivity and 90.91% specificity (Fig. 3B). Moreover, when we merged samples in Cohorts I and II to form an integral cohort and measured the expression of miR-185-5p in the HD and AA groups, we found that the expression of miR-185-5p was still significantly upregulated in the AA group (Fig. 3C). The AUC value of miR-185-5p (0.702) for diagnosing AA was also similar in Cohorts I and II (Fig. 3D). Further, univariate and multivariate analyses revealed that the expression of miR-185-5p was an independent diagnostic factor for colorectal AA (Additional file 9: Table S3).

### EV-delivered miR-185-5p is a potential biomarker for early-stage and advanced CRC

**Fig. 4:**
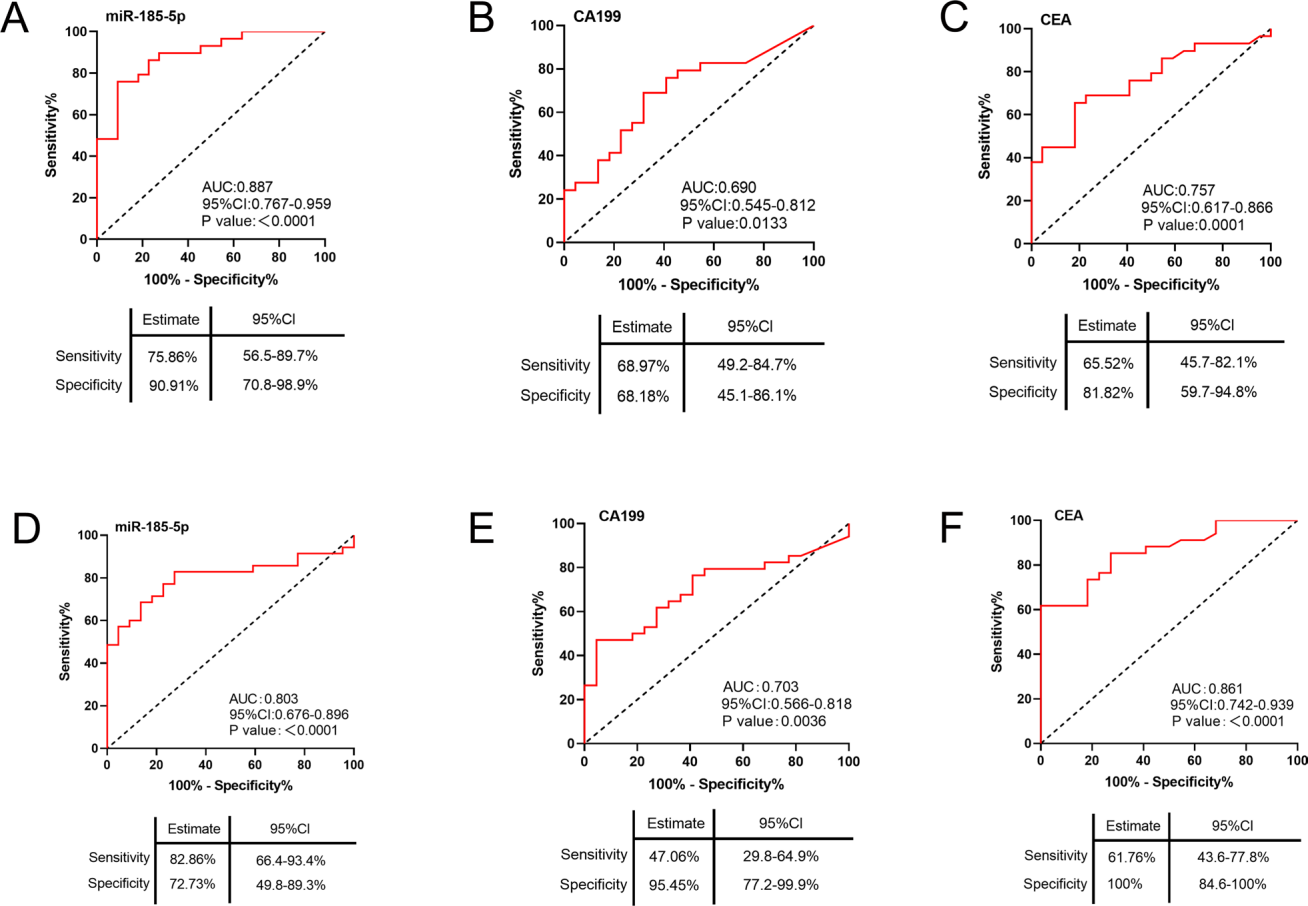
EV-delivered miR-185-5p is a potential biomarker for early-mid stage and advanced CRC. ROC curve analysis for **A** EV-delivered miR-185-5p **B** CEA **C** CA199 as parameters to discriminate early-mid stage CRC from HD in Cohort I. ROC curve analysis for **D** EV-delivered miR-185-5p **E** CEA **F** CA199 as parameters to discriminate advanced CRC from HD in Cohort II

The expression of EV-delivered miR-185-5p was significantly upregulated in early-mid stage and advanced CRC groups vs. AA group (Fig. 2E); therefore, we further evaluated whether miR-185-5p was a more beneficial biomarker in the diagnosis of early-mid stage and advanced CRC compared with existing biomarkers CA199 and CEA. In cohort I, the AUC value of miR-185-5p was 0.887 for the diagnosis of I-II stage CRC (Fig. 4A), which was significantly higher than that of CA199 (0.69, P = 0.0334) as revealed by significance analysis (Fig. 4B). CEA had an AUC of 0.757 with 65.52% sensitivity and 81.82% specificity. The AUC levels of miR-185-5p and CEA were similar (P = 0.0750) but CEA had low sensitivity and specificity compared with miR-185-5p (Fig. 4C). Furthermore, the expression of miR-185-5p was also correlated to the diagnosis of I-II stage CRC in the whole cohort (I + II, Additional file 10: Table S4), indicating that miR-185-5p was a potential independent factor for the diagnosis of I-II stage CRC.

Serum CEA and CA199 values are clinically used in the diagnosis of advanced CRC [19]; therefore, we further investigated whether miR-185-5p could also be used for the diagnosis of advanced CRC. Our results revealed that the AUC value of miR-185-5p for the diagnosis of III-IV stage CRC was 0.803 (Fig. 4D), which was similar to that of CA199 (0.703, P = 0.2066, Fig. 4E) and CEA (0.861, P = 0.5290, Fig. 4F). However, the diagnostic sensitivity of miR-185-5p was 82.86%, which was significantly higher than that of CA199 (47.06%) and CEA (61.76%). Moreover, the expression of miR-185-5p was correlated to the diagnosis of III-IV stage CRC in the whole cohort (I + II, Additional file 11: Table S5), indicating that miR-185-5p was a potential independent factor for the diagnosis of III-IV stage CRC.

Next, we evaluated the diagnostic efficacy of miR-185-5p in distinguishing early-mid stage and(or) advanced CRC from AA to trace the progression from colorectal AA to CRC. Although the AUC values among miR-185-5p (0.700), CA199 (0.677, P = 0.8388), and CEA (0.626, P = 0.4933) for the diagnosis of I-II stage CRC vs. AA were similar, the diagnostic sensitivity of miR-185-5p (75.86%) was not significantly lower than that of CA199 (79.31%) and was higher than that of CEA (51.72%) (Additional file 5: Fig. S5A-S5C). For the diagnosis of III-IV stage CRC vs. AA, the AUC value of miR-185-5p (0.631) was relatively lower but not significantly different from that of CA199 (0.681, P = 0.6706; significance analysis) and CEA (0.793, P = 0.0762; significance analysis) (Additional file 5: Fig. S5D-S5F). Overall, these results indicated that EV-delivered miR-185-5p was a potential independent factor for the diagnosis of both early-mid stage and advanced CRC. Simultaneously, miR-185-5p was a useful biomarker for the diagnosis of CRC vs. AA in addition to CA199 and CEA, indicating its potential as a continuous biomarker to trace disease progression from the normal state to AA and further to CRC.

### Upregulated miR-185-5p promotes the malignant progression of CRC

Given the fact that miR-185-5p-enriched EVs were present in the plasma of patients with CRC, we determined whether these EVs were derived from tumor cells. We confirmed the expression of miR-185-5p in the tumor tissues compared with adjacent normal tissues. The results showed that the expression of miR-185-5p was higher in tumor tissues than in adjacent normal tissues in 19/28 pairs of tissues (Additional file 6: Fig.S6A). Next, we measured the intracellular expression of miR-185-5p in four CRC cell lines and a normal colon epithelial cell line (FHC). We found that the expression of miR-185-5p in the CRC cell lines was higher than that in the FHC cell line (Additional file 6: Fig. S6B). We also checked miR-185-5p concentration in EVs which were harvested from the corresponding culture medium of the cell lines. Similar to our observations in the clinical samples, we confirmed the upregulated expression of miR-185-5p in the EVs from the culture supernatant of CRC cell lines (Additional file 6: Fig. S6C). The results of the cell proliferation assay of miR-185-5p revealed that elevated endogenous expression of miR-185-5p promoted the proliferation in CRC cell lines (Additional file 6: Fig. S6D).

**Fig. 5:**
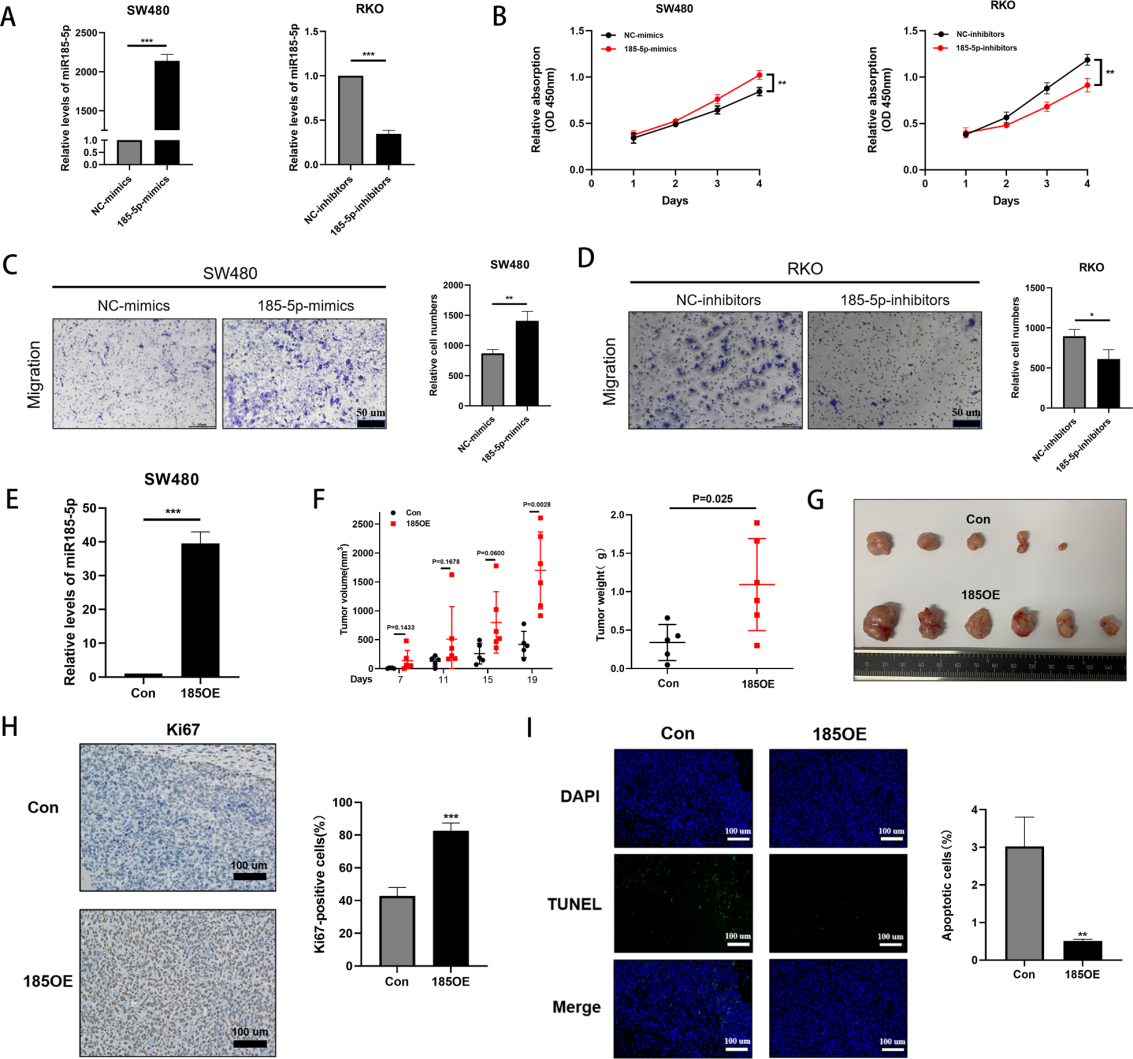
Upregulated miR-185-5p promotes the malignancy of CRC. **A** Detection of miR-185-5p expression in the SW480 cells transfected with miR-185-5p mimics and in the RKO cells transfected with miR-185-5p inhibitors using RT-qPCR. **B** An increase in the expression of miR-185-5p in the SW480 cells and a knockdown of the expression of miR-185-5p in the RKO cells affected their proliferation (CCK-8 assay). **C** An increase in the expression of miR-185-5p enhances the migration ability of the SW480 cells. **D** An Inhibition of the expression of miR-185-5p relieved the migration ability of the RKO cells. **E** Construction of the SW480 cells that stably overexpressed miR-185-5p. **F** Tumor volume and weight in the SW480-185OE group were larger than those in the SW480-Con group. **G** Comparison of the tumors taken from 185OE and Con groups. **H** Ki67-positive cells in tumors detected using immunohistochemical staining. **I** Tunel-positive cells in tumors detected using immunofluorescence staining. *, P < 0.05; **, P < 0.01; ***, P < 0.001. *, P < 0.05; **, P < 0.01; ***, P < 0.001

We attempted to confirm whether upregulated expression of miR-185-5p could promote the malignancy of human CRC cells. The RKO cell line showed a relatively higher endogenous miR-185-5p expression among the CRC cell lines, whereas the SW480 cell line showed a relatively lower endogenous miR-185-5p expression (Additional file 6: Fig.S6B). Therefore, we knocked down the expression of miR-185-5p in the RKO cells by transfecting miR-185-5p inhibitors. Next, we increased the expression of miR-185-5p in the SW480 cells by transfecting miR-185-5p mimics for functional assays (Fig. 5A and Additional file 6: Fig. S6B). After identifying the upregulation or downregulation of miR-185-5p in the relevant subclone cell lines, we first performed the CCK-8 proliferation assay and found that the increased expression of miR-185-5p in the SW480 cells could enhance their proliferation. In contrast, inhibition of miR-185-5p expression in the RKO cells could inhibit cell proliferation (Fig. 5B). Next, transwell assay showed that an increase in the expression of miR-185-5p promoted the migration of CRC cells (Fig. 5C), whereas inhibition of miR-185-5p expression repressed tumor cell migration (Fig. 5D). For in vivo assays, we first constructed SW480 cells that stably overexpressed miR-185-5p (SW480-185OE cells vs. SW480-Con cells as control) (Fig. 5E). Next, the SW480 cell line tumor-bearing mouse model was constructed. After tumor formation, we measured the tumor volume every four days. The tumor volume measurements from day 7 to 19 revealed that the volume and weight of subcutaneous tumors in the 185OE group were significantly larger than those in the Con group (Fig. 5F, G). Immunohistochemical staining also demonstrated that overexpression of miR-185-5p enhanced Ki67-positive cells (Fig. 5H) and reduced tunel-positive cells (Fig. 5I). These results indicated that EV-delivered miR-185-5p may be derived from tumors cell and the expression level of miR-185-5p was positively related to the malignancy of CRC.

## Discussion

The transformation from advanced adenoma to the colorectal tumor is the main cause of CRC, and the discovery of biomarkers that can be used for early diagnosis of AA and CRC has great clinical applications. However, such biomarkers, specifically specific continuous tracing markers, are limited for the diagnosis of both colorectal AA and CRC. In this study, we screened and verified EV-delivered miR-185-5p as a novel biomarker for the diagnosis of AA and CRC. We observed that the expression of miR-185-5p was significantly upregulated in AA vs. HD and CRC vs. AA groups. AUC values and logistic regression analysis revealed that tumor-derived miR-185-5p was a potential biomarker for the diagnosis of AA and also for the diagnosis of both early-mid stage and advanced CRC in addition to CA199 and CEA. Further, our data indicated that the elevated the expression of miR-185-5p promoted the malignancy of CRC, thereby suggesting an oncogenic function of miR-185-5p in CRC.

Although several EV-delivered miRNAs and free proteins have potential diagnostic value for AA in preclinical studies, a universally applicable circulating biomarker is not available for the clinical diagnosis of colorectal AA [20–22]. Here, we screened and identified that EV-delivered miR-185-5p was a novel and independent factor for the diagnosis of colorectal AA, as indicated by a high correlation between miR-185-5p expression and risk of AA (P = 0.002, Additional file 9: Table S3). EV-delivered miR-21, miR-29a, and miR-92a [23], as well as serum ALDH1B1 protein [24], can also be potentially used in the diagnosis of colorectal AA. However, the AUC values of EV-delivered miR-21, miR-29a, and miR-92a for the AA (vs. HD) were 0.687, 0.534, and 0.507 respectively [23], and these values were lower than that of miR-185-5p (Fig. 3). Although the AUC value of serum ALDH1B1 for the diagnosis of AA (vs. HD) was similar to that of miR-185-5p, it cannot be used as a continuously monitored biomarker to distinguish CRC from AA [24]. Therefore, plasma EV-delivered miR-185-5p may be a more favorable biomarker for the diagnosis of colorectal AA and monitor continuous disease progression.

EV-delivered miR-125a-3p and miR-150-5p are useful in the diagnosis of early-mid stage CRC [25, 26]. However, in comparison to them, miR-185-5p showed a higher AUC value (0.887 for miR-185-5p vs. 0.685 and 0.736 for miR-125a and miR-150-5p, respectively) and specificity (90.91% for miR-185-5p vs. 59.5% for miR-150-5p); the sensitivity between miR-185-5p and miR-150-5p was similar (75.86% for miR-185-5p vs. 59.5% for miR-150-5p). These results indicated that miR-185-5p was a potential biomarker for the diagnosis of early-mid stage CRC. However, the AUC values of miR-125a-3p and miR-150-5p in the diagnosis of early-mid stage CRC were not compared with CEA and CA199. Furthermore, in our study, the AUC value of miR-185-5p was significantly higher than that of CA199 (0.69, P = 0.0334). Although we observed a similar AUC of miR-185-5p and CEA, sensitivity (65.52% for CEA vs. 75.86% for miR-185-5p) and specificity (81.82% for CEA vs. 90.91% for miR-185-5p) were lower when CEA was considered for the diagnosis of early-mid stage CRC. EV-delivered miR-1229 and miR-25-3p are the available biomarkers for the diagnosis of advanced CRC [27]. The AUC value of miR-185-5p was similar to that of miR-1229 and miR-25-3p. However, their diagnostic efficacy has not been reported in early-mid stage CRC. Overall, miR-185-5p may be a potential biomarker for the diagnosis of CRC.

The circulating diagnostic biomarkers that can continuously monitor the progression from AA to early-mid stage and advanced CRC have immense clinical applications. Although serum CEA and CA199 have great advantages in the diagnosis of advanced CRC (especially in stage IV CRC), their diagnostic efficiency in early-mid stage CRC is poor [7]. We observed that the expression of several miRNAs was upregulated in AA and early-mid stage CRC but downregulated in advanced stages of CRC (such as miR-320d, data not shown). Therefore, miRNAs that exhibit fluctuating profiles are not conducive to continuously trace the disease progression. The expression of miR-185-5p was stably upregulated during the progression from AA to tumorigenesis (stages I–II) and advanced stage (stages III–IV) (Fig.S4). This indicated that miR-185-5p may be a novel biomarker for the diagnosis of AA and can be a potential biomarker to diagnose early-mid stage and advanced CRC in addition to CEA and CA199.

## Conclusion

Our results indicated that EV-delivered miR-185-5p could serve as a biomarker for continuously tracing the transition from precancerous lesions (AA) to tumorigenesis (CRC stages I–II) and further to tumor progression (CRC stages III–IV). Our findings also suggested that miR-185-5p-enriched EVs might be derived from tumor cells and the expression level of miR-185-5p was positively related to the malignancy of CRC. A detailed mechanistic study with an extended validation cohort of patients from China, other Asian countries, and western countries will further consolidate our findings.

## Supplementary Information

**Additional file 1: Figure S1. **Biological characteristics of EVs isolated from plasma. (A) TEM images showing round or oval-shaped plasma EVs without the nucleus in HD, AA, and CRC groups. (B) The expression of EV-specific markers and negative marker was detected in EVs isolated from plasma among HD, AA, and CRC groups by western blotting. (C) NTA showing the particle size range and concentration of EVs in HD, AA, and CRC groups. Data from one of the HD, AA or CRC cases were showed as representative example respectively. (D) The comparison of diameter and concentration of plasma derived EV among HD, AA and CRC group. Data were presented by Mean ± SD. (E) Box plot comparing plasma total RNA and EV RNA concentrations among HD, AA, and CRC groups.

**Additional file 2: Figure S2. **Cluster analysis of top 25 different miRNAs among different groups. Heatmap showing top 25 differentially expressed EV-delivered miRNAs between (A) HD and AA, (B) HD and CRC, and (C) AA and CRC groups (screened using miRNA deep sequencing assay).

**Additional file 3: Figure S3. **Study design.

**Additional file 4: Figure S4. **Relative expression of miR-185-5p between patients with early-mid stage and advanced CRC in the whole cohort (I + II).

**Additional file 5: Figure S5. **EV-delivered miR-185-5p is a potential biomarker for differentiating early-mid stage and advanced CRC from AA. ROC curve analyses for (A) EV- delivered miR-185-5p, (B) CEA, and (C) CA199 as parameters to differentiate early-mid stage CRC from AA in Cohort I. ROC curve analyses for (D) EV-delivered miR-185-5p, (E) CEA, and (F) CA199 as parameters to differentiate advanced CRC from AA in Cohort II.

**Additional file 6: Figure S6. **EV-delivered miR-185-5p may be released from the tumor, and the miR-185-5p levels correlate with the proliferation of CRC cells. (A) Levels of miR-185-5p the in the tumor tissue were significantly higher than that in the adjacent normal tissue. (B) Intracellular expression of miR-185-5p in normal colon epithelial cell line FHC vs. four CRC cell lines. (C) Expression of miR-185-5p in EVs in normal colon epithelial cell line FHC vs. four CRC cell lines. (D) TEM images showing round or oval-shaped EVs without the nucleus extracted from the cell media. The image showed one representative example. (E) 48-h cell proliferation assay performed using FHC and four CRC cell lines. *, P＜0.05；**，P＜0.01；***，P＜0.001.

**Additional file 7. Table S1.** The clinical and pathologic characteristics of the patients.

**Additional file 8. Table S2.** 10 candidate miRNAs that were upregulated or downregulated in both AA vs. HD and CRC vs. AA groups.

**Additional file 9: Table S3.** Univariate and multivariate analyses of the association of predictors with advanced adenoma (I+II cohort).

**Additional file 10: Table S4.** Univariate and multivariate analyses of the association of predictors with early-mid stage CRC (I+II cohort).

**Additional file 11: Table S5.** Univariate and multivariate analyses of the association of predictors with advanced CRC (I+II cohort).

## Data Availability

All data generated or analyzed during this study were included either in this article methods section. Other data that support the findings of this study are available from the corresponding author upon reasonable request.

## Abbreviations

CRC: Colorectal cancer
AA: Advanced adenoma
HD: Healthy donors
AUC: Receiver operating characteristic curve
EV: Extracellular vesicle
NTA: Nanoparticle tracking analysis
TEM: Transmission electron microscopy
CEA: Carcinoembryonic antigen
CA199: Carbohydrate antigen 199
