# Supplementary figures and images for: Discovery of extracellular vesicle-delivered miR-185-5p in the plasma of patients as an indicator for advanced adenoma and colorectal cancer
Source: J Transl Med. 2023 Jun 29;21:421. doi: 10.1186/s12967-023-04249-6 (PMC10308673; doi:10.1186/s12967-023-04249-6)

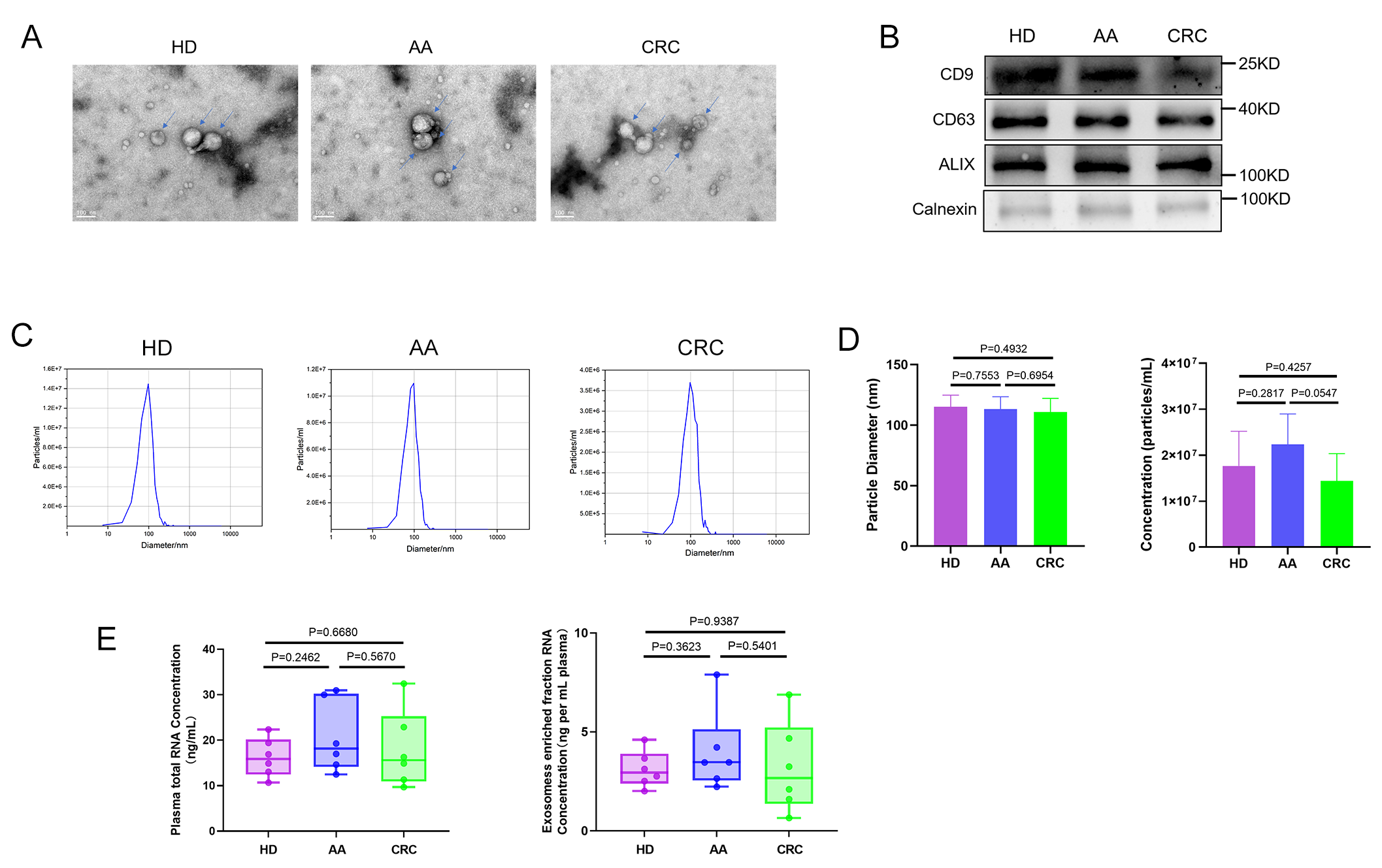

Supplement: Supplementary file 1 — Additional file 1: Figure S1. Biological characteristics of EVs isolated from plasma. (A) TEM images showing round or oval-shaped plasma EVs without the nucleus in HD, AA, and CRC groups. (B) The expression of EV-specific markers and negative marker was detected in EVs isolated from plasma among HD, AA, and CRC groups by western blotting. (C) NTA showing the particle size range and concentration of EVs in HD, AA, and CRC groups. Data from one of the HD, AA or CRC cases were showed as representative example respectively. (D) The comparison of diameter and concentration of plasma derived EV among HD, AA and CRC group. Data were presented by Mean ± SD. (E) Box plot comparing plasma total RNA and EV RNA concentrations among HD, AA, and CRC groups. [file 12967_2023_4249_MOESM1_ESM.tif]

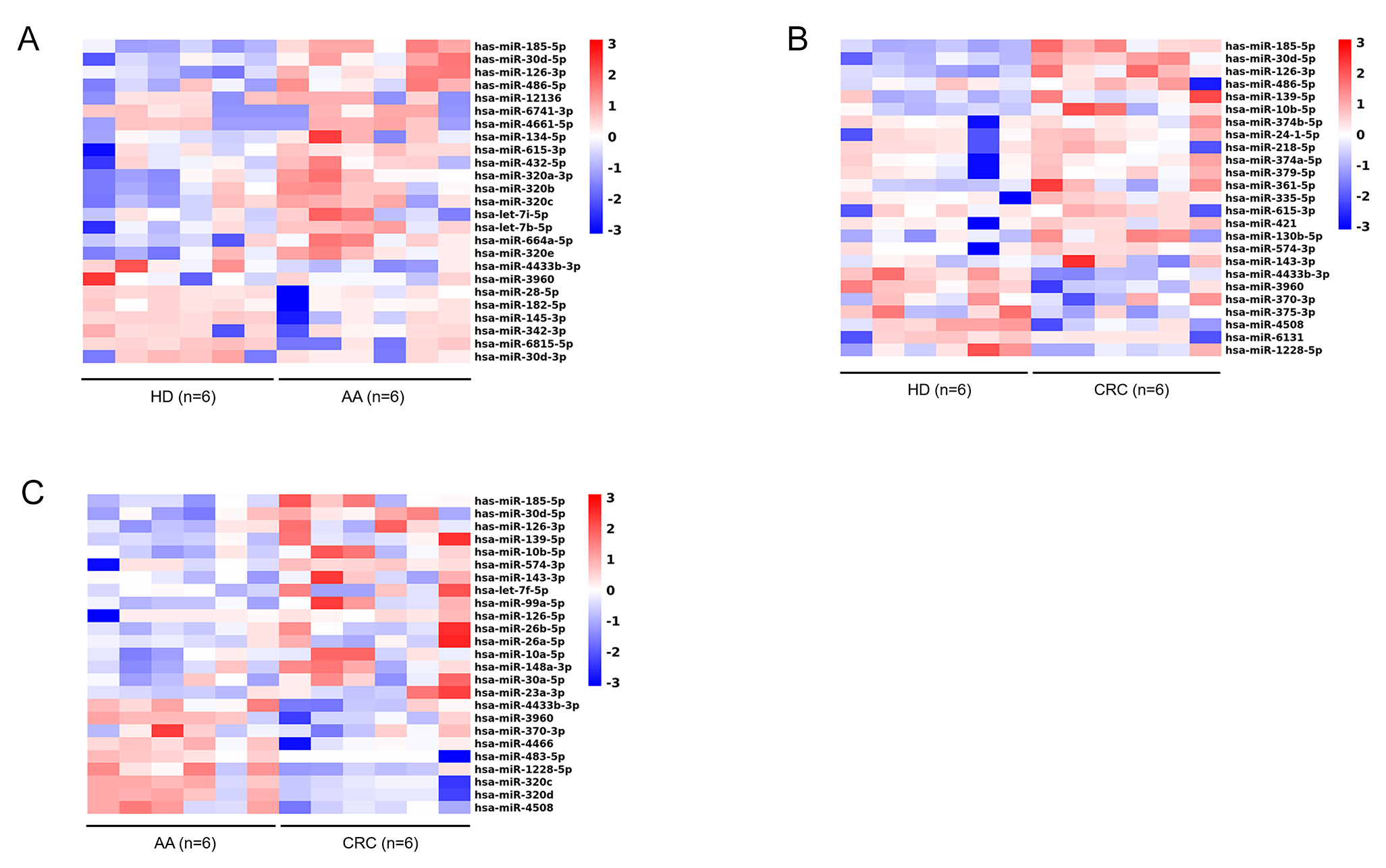

Supplement: Supplementary file 2 — Additional file 2: Figure S2. Cluster analysis of top 25 different miRNAs among different groups. Heatmap showing top 25 differentially expressed EV-delivered miRNAs between (A) HD and AA, (B) HD and CRC, and (C) AA and CRC groups (screened using miRNA deep sequencing assay). [file 12967_2023_4249_MOESM2_ESM.tif]

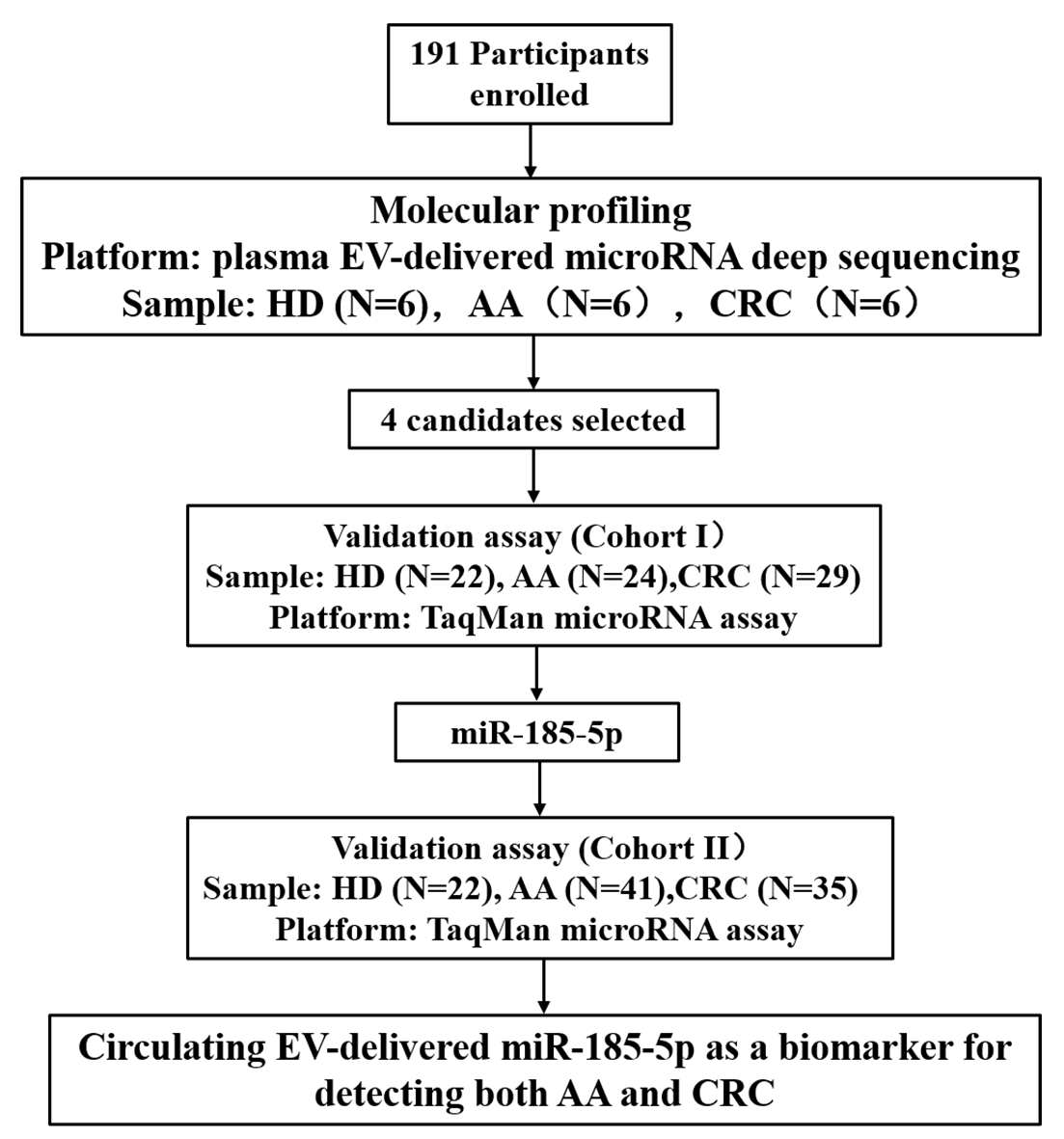

Supplement: Supplementary file 3 — Additional file 3: Figure S3. Study design. [file 12967_2023_4249_MOESM3_ESM.tif]

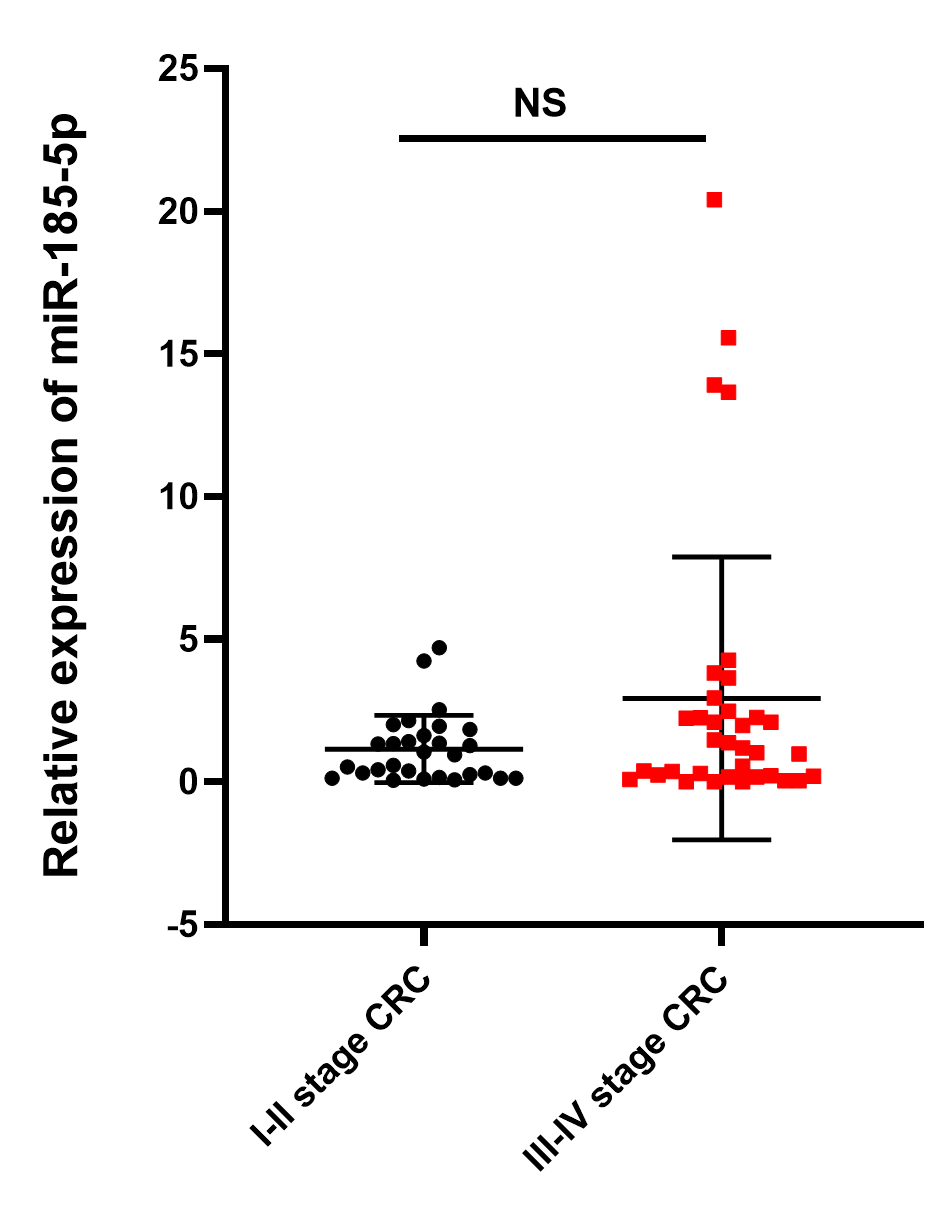

Supplement: Supplementary file 4 — Additional file 4: Figure S4. Relative expression of miR-185-5p between patients with early-mid stage and advanced CRC in the whole cohort (I + II). [file 12967_2023_4249_MOESM4_ESM.tif]

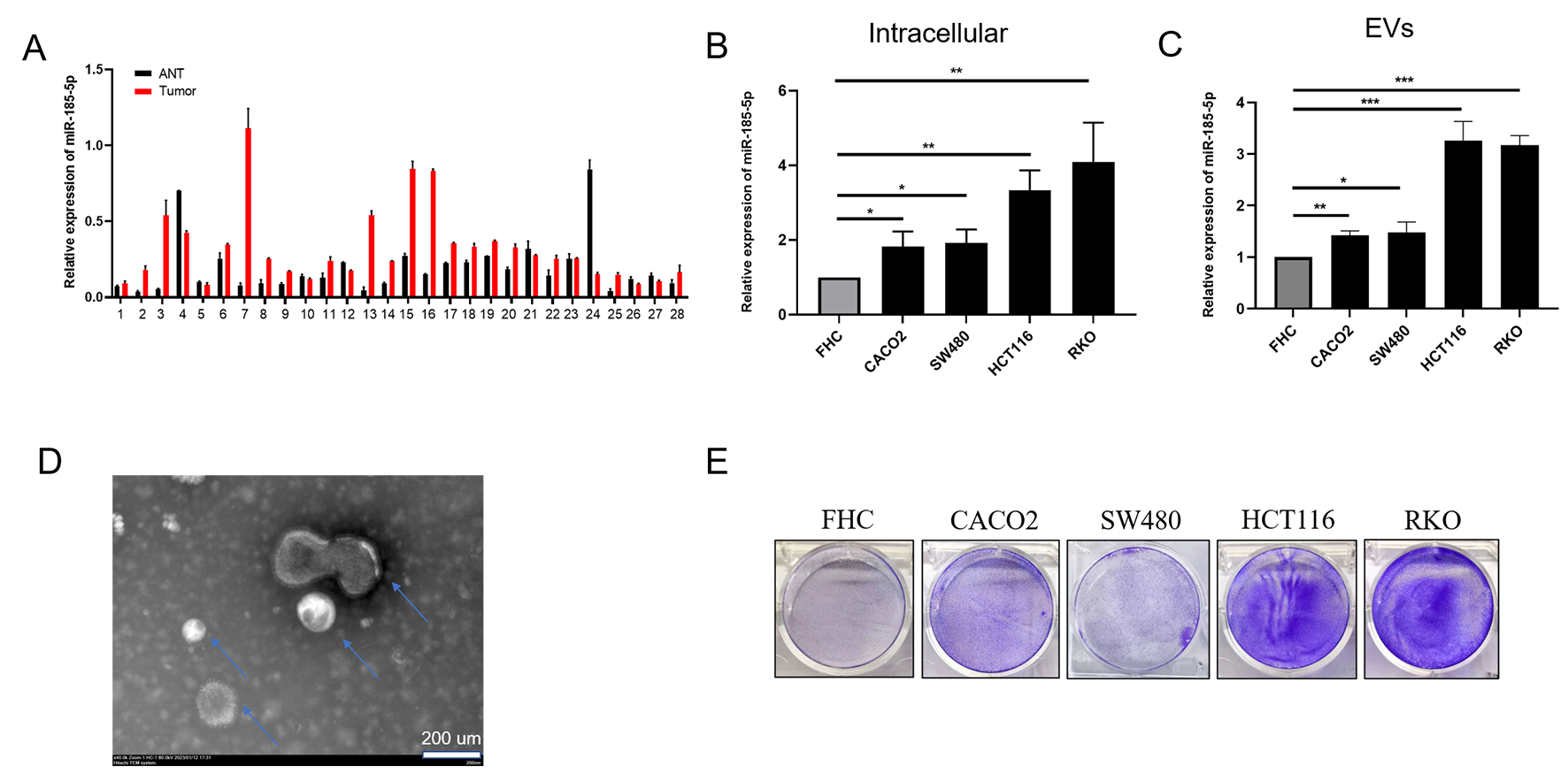

Supplement: Supplementary file 5 — Additional file 5: Figure S5. EV-delivered miR-185-5p is a potential biomarker for differentiating early-mid stage and advanced CRC from AA. ROC curve analyses for (A) EV- delivered miR-185-5p, (B) CEA, and (C) CA199 as parameters to differentiate early-mid stage CRC from AA in Cohort I. ROC curve analyses for (D) EV-delivered miR-185-5p, (E) CEA, and (F) CA199 as parameters to differentiate advanced CRC from AA in Cohort II. [file 12967_2023_4249_MOESM5_ESM.tif]
