# Supplementary material for: Discovery of extracellular vesicle-delivered miR-185-5p in the plasma of patients as an indicator for advanced adenoma and colorectal cancer
Source: J Transl Med. 2023 Jun 29;21:421. doi: 10.1186/s12967-023-04249-6 (PMC10308673; doi:10.1186/s12967-023-04249-6)
Supplement: Supplementary file 9 — Additional file 9: Table S3. Univariate and multivariate analyses of the association of predictors with advanced adenoma (I+II cohort). [file 12967_2023_4249_MOESM9_ESM.docx]

**Table S3. Univariate and multivariate analyses of the association of predictors with advanced adenoma (I+II cohort)**

| Parameters | Total  (n) | Univariate analysis | | Multivariate analysis | |
| --- | --- | --- | --- | --- | --- |
|  |  | OR (95%CI) | P value | OR (95%CI) | P value |
| Age |  |  |  |  |  |
| ≤50 | 36 |  |  |  |  |
| ＞50 | 73 | 1.81 (0.80-4.06) | 0.1520 |  |  |
| Gender |  |  |  |  |  |
| Female | 50 |  |  |  |  |
| Male | 59 | 2.90 (1.31-6.40) | 0.0084 | 2.90 (1.24-6.79) | 0.0143 |
| EV-delivered  miR-185-5p expression |  |  |  |  |  |
| Low | 71 |  |  |  |  |
| High | 38 | 6.14 (2.28-16.51) | 0.0003 | 6.14 (2.23-16.94) | 0.0002 |
