# Supplementary material for: Discovery of extracellular vesicle-delivered miR-185-5p in the plasma of patients as an indicator for advanced adenoma and colorectal cancer
Source: J Transl Med. 2023 Jun 29;21:421. doi: 10.1186/s12967-023-04249-6 (PMC10308673; doi:10.1186/s12967-023-04249-6)
Supplement: Supplementary file 10 — Additional file 10: Table S4. Univariate and multivariate analyses of the association of predictors with early-mid stage CRC (I+II cohort). [file 12967_2023_4249_MOESM10_ESM.docx]

**Table S4. Univariate and multivariate analyses of the association of predictors with early-mid stage CRC (I+II cohort)**

| Parameters | Total  (n) | Univariate analysis | | Multivariate analysis | |
| --- | --- | --- | --- | --- | --- |
|  |  | OR (95%CI) | P value | OR (95%CI) | P value |
| Age |  |  |  |  |  |
| ≤60 | 55 |  |  |  |  |
| ＞60 | 18 | 2.05 (0.71-5.90) | 0.185 |  |  |
| Gender |  |  |  |  |  |
| Female | 43 |  |  |  |  |
| Male | 30 | 0.63 (0.24-1.66) | 0.353 |  |  |
| CEA |  |  |  |  |  |
| Low (＜5) | 67 |  |  |  |  |
| High (≥5) | 6 | 3.36 (0.57-19.69) | 0.997 |  |  |
| CA199 |  |  |  |  |  |
| Low (＜37) | 69 |  |  |  |  |
| High (≥37) | 4 | 4.96 (0.49-50.24) | 0.175 |  |  |
| EV-delivered miR-185-5p expression |  |  |  |  |  |
| Low | 44 |  |  |  |  |
| High | 29 | 19.9 (5.9-66.8) | ＜0.0001 | 22.31(5.66-87.98) | ＜0.0001 |
