# Supplementary material for: Discovery of extracellular vesicle-delivered miR-185-5p in the plasma of patients as an indicator for advanced adenoma and colorectal cancer
Source: J Transl Med. 2023 Jun 29;21:421. doi: 10.1186/s12967-023-04249-6 (PMC10308673; doi:10.1186/s12967-023-04249-6)
Supplement: Supplementary file 11 — Additional file 11: Table S5. Univariate and multivariate analyses of the association of predictors with advanced CRC (I+II cohort). [file 12967_2023_4249_MOESM11_ESM.docx]

**Table S5.** Univariate and multivariate analyses of the association of predictors with advanced CRC (I+II cohort)

| Parameters | Total  (n) | Univariate analysis | | Multivariate analysis | |
| --- | --- | --- | --- | --- | --- |
|  |  | OR (95%CI) | P value | OR (95%CI) | P value |
| Age |  |  |  |  |  |
| ≤60 | 50 |  |  |  |  |
| ＞60 | 29 | 6.75 (2.43-18.75) | 0.0002 | 13.70(2.28-82.33) | 0.0042 |
| Gender |  |  |  |  |  |
| Female | 40 |  |  |  |  |
| Male | 39 | 2.69 (1.08-6.72) | 0.034 | 9.99(1.71-58.43) | 0.0106 |
| CEA |  |  |  |  |  |
| Low (＜5) | 60 |  |  |  |  |
| High (≥5) | 19 | 2.20 (1.51-3.20) | 0.998 |  |  |
| CA199 |  |  |  |  |  |
| Low (＜37) | 68 |  |  |  |  |
| High (≥37) | 11 | 17.2(2.08-142.4) | 0.0083 | 37.5(2.15-655.01) | 0.013 |
| EV-delivered miR-185-5p expression |  |  |  |  |  |
| Low | 44 |  |  |  |  |
| High | 35 | 16.43(5.33-50.71) | ＜0.0001 | 12.33(2.95-51.61) | 0.0006 |
